# Developing an image-based grading scale for peripheral drusen to investigate associations of peripheral drusen type with age-related macular degeneration

**DOI:** 10.1038/s41598-024-70352-3

**Published:** 2024-08-29

**Authors:** Paripoorna Sharma, Fritz Gerald P. Kalaw, Andrew Lin, Evan H. Walker, Shyamanga Borooah

**Affiliations:** 1 University of California, San Diego, La Jolla, USA; 2 Jacobs Retina Center, University of California San Diego, La Jolla, USA; 3 Shiley Eye Institute, University of California, San Diego, 9415 Campus Point Drive, La Jolla, CA 92093 USA

**Keywords:** Age-related macular degeneration, Drusen, Peripheral drusen, Peripheral retinal abnormalities, Retinal imaging, Ultra-widefield fundus imaging, Eye abnormalities, Retinal diseases

## Abstract

Age-related macular degeneration (AMD) is a leading cause of blindness. It is associated with peripheral drusen which has not been categorized. We investigated peripheral drusen to validate an image grading system and to understand possible associations between peripheral drusen and AMD. We collated clinical data, ultra-widefield (UWF) pseudocolor fundus images and Spectral-Domain Optical Coherence Tomography (SD-OCT) scans from consecutive retinal patients. SD-OCT scans were used to determine AMD stage. A masked retinal specialist recorded the types of peripheral drusen observed in UWF images. Eyes whose UWF images did not pass quality screening and those without AMD and peripheral drusen were excluded from the study. Statistical tests were utilized to determine the validity of our grading system and associations of peripheral drusen with AMD. A total of 481 eyes (283 subjects) were included in the study (mean age 73.1 ± 1.2years, 64.3% female). Interobserver and test–retest statistical analyses to evaluate the UWF image grading system resulted in Cohen’s Kappa 0.649 (*p* < 0.001) and 0.922 (*p* < 0.001) respectively. A total of 284 (59.0%), 28 (5.8%), 15 (3.1%), 22 (4.6%), 4 (0.8%), 39 (8.1%), and 32 (6.7%) eyes had hard, soft, reticular, cuticular, atrophic, mixed drusen, and mixed drusen and atrophy respectively in at least one peripheral retinal quadrant. Hard peripheral drusen was significantly associated with the presence of AMD (*p* = 0.010). Peripheral drusen types were variably seen in retinal patients with and without AMD. We validated a peripheral drusen grading system and provided an image library to assist in the identification of peripheral drusen. Our study found an association between peripheral hard drusen and an AMD diagnosis but did not find a link between peripheral drusen and severity of AMD.

## Introduction

Age-related macular degeneration (AMD) is a leading cause of blindness resulting from damage to the macula. While AMD is commonly associated with drusen deposits accumulating in the macula, recent studies have suggested that AMD is also associated with the presence of peripheral retinal drusen^1,2^.

Drusen result from extracellular debris accumulating below the retinal pigment epithelial (RPE) resulting from a number of disease processes^3,4^. Numerous studies have investigated macular drusen, which develop as part of aging and as part of AMD^3,5–7^. Macular drusen have been linked to a decrease in visual acuity and quality of life in AMD^8^. Grading scales have identified that the size and type of macular drusen can be used to predict progression of AMD^9^. While there is little evidence that peripheral drusen directly affect vision, studying peripheral drusen may provide new insights into AMD, because of the reported association of peripheral drusen with this disease. Past studies have found evidence that peripheral drusen may have some differences from macular drusen in terms of morphology, related genotypes, and physical composition^4,10^. Studying peripheral drusen would potentially shed more light on the effects of photooxidative stress and photoreceptor composition in the retinal periphery, potentially improving our understanding of macular changes in AMD.

As UWF imaging becomes more widely used in clinical settings, studies have begun to examine peripheral drusen in more detail. Some studies have shown that different types of peripheral drusen are seen commonly in patients with and without AMD^11,12^. Currently, relatively little has been described about the types and prevalence of peripheral drusen seen in patients. In addition, it is unclear whether peripheral drusen is a prognostic marker for AMD severity.

The present study aims to develop a grading scale for the types of peripheral drusen in patients with AMD. Our hypothesis is that sub-types of peripheral drusen have a stronger association with AMD and its progression, similar to macular drusen in AMD. The rationale for doing these studies is that, to our knowledge, there has not yet been a report investigating the type of peripheral drusen and AMD, unlike for macular drusen and if an association was found, it may be an important peripheral marker for AMD progression. As a result, we also aim to study any association between the prevalence of different peripheral drusen types with AMD and stage of AMD to determine whether any relationships exist.

## Methods

This study was approved by the Institutional Review Board at the University of California–San Diego, California, USA. This study complied with the Health Insurance Portability and Accountability Act of 1996 and written informed patient consent was obtained by the institution’s protocol. Data was anonymized for patient safety and all collection and analysis was conducted by abiding with the Principles of the Declaration of Helsinki.

We performed a retrospective, observational clinical study. Consecutive retinal patients that visited two retinal specialists at the Shiley Eye Institute, University of California, San Diego (UCSD) between 1st January and 29th December 2021 were included in the study. Both specialists use UWF pseudocolor fundus imaging (Optos P200DTx, Optos plc, Dunfermline, UK) and spectral-domain Optical Coherence Tomography (SD-OCT) for all patients. The patients followed the standard clinic imaging protocol. During their visit, each patient was first dilated with 1 drop of 1% tropicamide and 2.5% phenylephrine in each eye. After a minimum wait time of 10 min, patients then underwent standard UWF pseudocolor imaging and SD-OCT scans by a trained medical photographer. The same day UWF pseudocolor fundus and SD-OCT images were collated from both eyes of each patient. The demographic data for each patient was also collected and included age, gender, and ethnicity.

### Image processing and grading

The UWF images were directly downloaded from the image database in JPEG format and processed to exclude the central 45° region of the retina centered on the fovea, using previously described methods^13,14^. Each remaining 90° section of the images was then divided into four quadrants: superior (S), nasal (N), inferior (I), and temporal (T) illustrated in Supplementary Fig. 1^2^.

The UWF images were then screened for quality. The imaging inclusion criteria required an unobstructed view of at least 60% of each quadrant. If this criterion was fulfilled, each UWF image was then examined for visibility of fourth order arterioles in each quadrant. Any images that fulfilled both criteria for the initial quality check were then forwarded for further analysis^14^.

A masked retinal specialist (FGW) determined the stage of AMD utilizing a modified Delphi technique outlined in Ferris et al^15^. by examining SD-OCT scans that passed initial UWF quality screening, sorted into the following staging categories: No AMD, early AMD, intermediate AMD, late AMD (general atrophy, GA), and late AMD (wet).

The different peripheral drusen phenotypes were identified by two retinal specialists on a pilot review of 40 UWF fundus images and generated reference images. A masked retinal specialist examined the images by quadrant and determined the types of peripheral drusen that could be recorded by using standard descriptions of macular drusen^16,17^. After reviewing the types of peripheral drusen we observed in the pilot images, we did not find a significant difference in the appearance of peripheral drusen when compared to the macular drusen literature. As such, the peripheral drusen categories included in the final study were hard drusen, soft drusen, reticular drusen, cuticular drusen, atrophic drusen, mixed drusen, and mixed drusen/atrophy (Supplementary Fig. 2).

A retinal specialist then recorded the type of peripheral drusen observed in UWF images by quadrant, using standard descriptions of the peripheral drusen image references^16,17^. Patients that had no diagnosis of AMD and no visible drusen in the peripheral retina on UWF imaging, that passed quality screening, were excluded from the study as these patients did not fit within the scope of the study. Eyes with UWF images that had visible peripheral laser marks, pan-retinal photocoagulation (PRP), and asteroid hyalosis were excluded to reduce confounding variables.

### Grading validation

To first validate our evaluation of the identification of peripheral drusen in the UWF images, an inter-observer validation was performed. A primary and secondary masked retinal specialist recorded peripheral drusen found in the periphery of each quadrant across a sample of 50 right eye and 50 left eye UWF images. Interrater reliability and agreement were evaluated using Cohen’s Kappa coefficients. The agreement analysis was conducted on a global and sectoral basis using the overall cohort data.

Additionally, a test–retest validation was used to assess reliability for the primary masked retinal specialist. The peripheral drusen identified in each quadrant of the UWF images was recorded for the same 100 images at two different time points by the primary masked retinal specialist, one week apart. The agreement analysis was also conducted using Cohen’s Kappa coefficients on a global and sectoral basis with the overall cohort data.

### Statistical analysis

Subject and eye-level demographic and clinical characteristics are presented as count (%) and mean (95% CI) for categorical and continuous parameters, respectively. A stratified analysis was conducted to evaluate the association between AMD status and Drusen findings using Generalized Linear Mixed-Effects (GLME) models. All mixed-effects models were fitted with a random intercept to adjust for within-subject variability to account for the correlation derived by the inclusion of two eyes from one patient.

GLME and Mixed Analysis of Variance (ANOVA) models were fit to test the association between race stratified groups with each drusen type. Two age groups were decided based on the median age, with one cohort below the calculated age median and the other above. We also utilized a cumulative link mixed model (CLMM) odds ratio to investigate the association between age and the magnitude of Drusen findings. The number of quadrants with Drusen was categorized ordinally and ranked from 0 to 4 affected quadrants. The model included random intercepts for individual patients, allowing for the assessment of age-related changes in the number of affected quadrants while accounting for variability between patients.

Statistical analyses were performed using R programming language for statistical computing, Version 4.3.2^18^. *P-*values less than 0.05 were considered statistically significant.

## Results

A total of 3342 UWF images of individual retina were initially reviewed from 1,671 consecutive patients seen in the retina clinics of UCSD Shiley Eye Institute during 2021. After initial UWF quality screening, 1461 (43.7%) eyes were excluded following the criteria outlined in the methods. Of the remaining 1881 (56.3%) eyes, a further 1388 eyes were excluded because these patients did not have any visible peripheral drusen in their UWF images and eyes were also not associated with an AMD diagnosis. Finally, 12 eyes (0.4%) were excluded due to other pathology including PRP, laser marks, and asteroid hyalosis identified in the periphery of the UWF images. The reasons for exclusion of eyes are summarized in Supplementary Table 1.

A total of 481 eyes across a total of 282 unique study subjects were included in the final analysis. The patients had a mean age of 73.1 ± 1.2 years. With 182 (64.3%) patients being female and 217 (76.7%) subjects self-identifying as Caucasian, 24 (8.5%) identifying as Asian, 13 (4.6%) identifying as Hispanic, and 5 (1.8%) self-identifying as African American or Black. All patient demographics are summarized in Table 1.

**Table 1:** Patient demographics summary.

Demographic category	Final cohort	Excluded subjects
	N = 283 Patients	N = 1388 Patients
Age	73.1 (71.9, 74.4)	60.2 (59.2, 61.3)
Race		
White	217 (76.7%)	742 (53.5%)
Asian	24 (8.5%)	230 (16.6%)
Hispanic	13 (4.6%)	128 (9.2%)
Other or Mixed Race	24 (8.5%)	156 (11.2%)
African American or Black	5 (1.8%)	63 (4.5%)
Unknown or Not Reported	0 (0.0%)	52 (3.7%)
Native Hawaiian or Other Pacific Islander	0 (0.0%)	7 (0.5%)
American Indian or Alaskan Native	0 (0.0%)	10 (0.7%)
Sex		
Female	182 (64.3%)	746 (53.7%)
Male	101 (35.7%)	642 (46.3%)

### Grading validation

As no previous grading systems had been identified for grading of peripheral drusen on UWF imaging we first attempted to develop a novel grading system initially modifying existing definitions for macular drusen. To validate this peripheral drusen grading system, an inter-observer agreement was initially performed by two retinal fellows who were masked to the others grading. The results from the inter-observer validation of 100 UWF images are summarized in Supplementary Table 2 and resulted in a global Kappa value of 0.649 with a *p* < 0.001. These values indicated a substantial level of inter-observer agreement in grading peripheral drusen^19^. To further validate the UWF grading system, we next performed a test–retest study. The results from the test–retest validation using 100 UWF images are summarized in Supplementary Table 3 and yielded a Kappa value of ranging from 0.922 with *p* < 0.001. These values indicated an almost perfect level of agreement^19^. Taken together, these preliminary analyses validated the use of our grading system to analyze peripheral drusen in UWF images.

### Prevalence of peripheral drusen

Having validated a grading system for peripheral drusen we first sought to understand the prevalence, type, and distribution of peripheral drusen.

Using our new grading system we next looked to find the prevalence of different types of peripheral drusen. This analysis looked to see how many times at least 1 druse of a certain type was found in an eye. In total, 355 (73.8%) eyes across 213 (75.3%) subjects had drusen in at least one quadrant with the remaining 126 (26.2%) eyes that were included in the study having a diagnosis of AMD with no peripheral drusen. A total of 284 (59.0%), 28 (5.8%), 15 (3.1%), 22 (4.6%), 4 (0.8%), and 39 (8.1%) eyes had hard, soft, reticular, cuticular, atrophic, and mixed peripheral drusen respectively in at least one quadrant. A total of 32 (6.7%) eyes had mixed drusen and atrophy in at least one quadrant. The distribution of peripheral drusen by quadrant is illustrated in Supplementary Fig. 3. There was no significant difference in the distribution of peripheral drusen across the four quadrants. Taken together, our findings suggest that drusen is a common feature in the periphery of retina and that prevalence increases with age.

### Relationship between peripheral drusen and AMD

**Figure 1:**
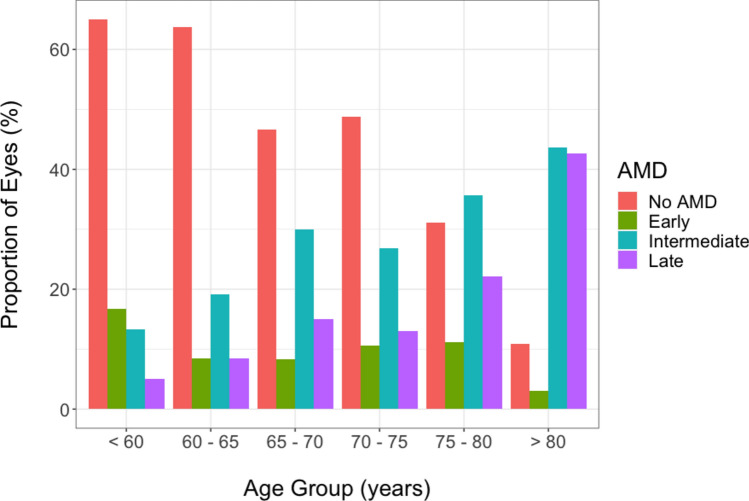
Graph depicting proportion of patients in each AMD stage by age cohort.

**Figure 2:**
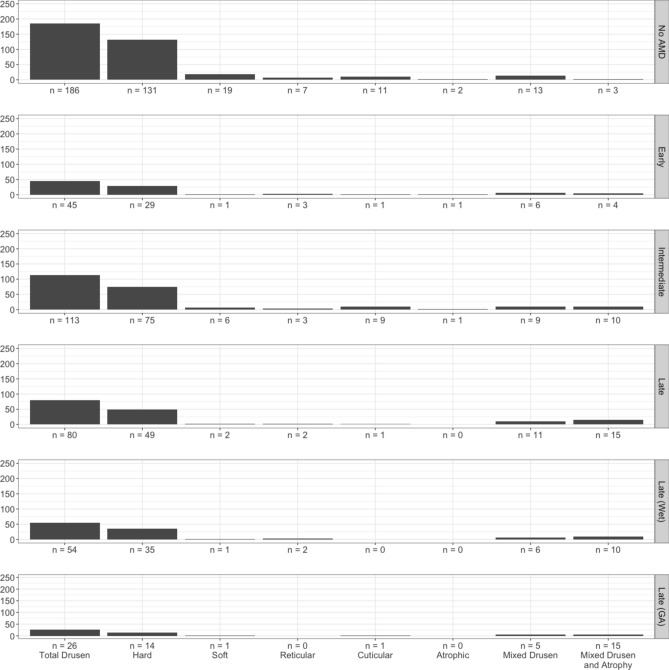
Graph depicting eyes observed with each type of peripheral drusen by AMD stage.

We next looked to see if there was a relationship between the presence of peripheral drusen and AMD, as seen in previous studies. Of the 355 eyes with peripheral drusen, a total of 163 eyes (45.9%) were observed without AMD, 34 eyes (9.6%) with early-stage AMD, 93 eyes (26.2%) with intermediate-stage AMD, and 65 eyes (18.3%) with late-stage AMD. Of the patients with late-stage AMD, 45 eyes (69.2%) were diagnosed with wet AMD and 20 eyes (30.8%) were GA. The distribution of AMD diagnosis by proportion of patient age is illustrated in Fig. 1. We then sought to understand whether peripheral drusen was a marker of AMD severity by looking to see if peripheral drusen type were associated with AMD stage. The prevalence of peripheral drusen by AMD stage is illustrated in Fig. 2. The prevalence of peripheral drusen by AMD stage, including late stage wet or atrophic types, is outlined in Supplementary Table 4. We found that there was no overall association between peripheral drusen and AMD grade in our cohort.

As we did not find an overall relationship between peripheral drusen presence and AMD or AMD severity in our cohort, we sought to investigate whether there was a significant association between AMD stage and peripheral drusen type as type of macular drusen is associated with progression of AMD at the macula. We found that hard drusen was significantly associated with the overall presence of AMD (*p* = 0.010) The GLME model was used to examine the association between AMD staging and peripheral drusen and is summarized in Table 2.

**Table 2:** The generalized linear mixed-effects model was used to generate *p-*values associated between AMD staging determined by SD-OCT and peripheral drusen findings found in UWF images.

	No AMD	Any AMD	*p*-value
	(n = 196 eyes)	(n = 284 eyes)	
Total drusen			
Not present	33 (16.8%)	93 (32.7%)	0.311
Present	163 (83.2%)	191 (67.3%)	
Hard			
Not present	65 (33.2%)	131 (46.1%)	**0.010**
Present	131 (66.8%)	153 (53.9%)	
Soft			
Not present	177 (90.3%)	275 (96.8%)	0.173
Present	19 (9.7%)	9 (3.2%)	
Reticular			
Not present	189 (96.4%)	276 (97.2%)	0.536
Present	7 (3.6%)	8 (2.8%)	
Cuticular			
Not present	185 (94.4%)	273 (96.1%)	0.857
Present	11 (5.6%)	11 (3.9%)	
Atrophic			
Not present	194 (99.0%)	282 (99.3%)	0.710
Present	2 (1.0%)	2 (0.7%)	
Mixed drusen			
Not present	183 (93.4%)	259 (91.2%)	0.205
Present	13 (6.6%)	25 (8.8%)	
Mixed drusen and atrophy			
Not present	193 (98.5%)	255 (89.8%)	0.389
Present	3 (1.5%)	29 (10.2%)	

Significant *p-values* are bolded and represent those that are ≤ 0.05.

### Relationship between peripheral drusen and demographic characteristics

We also examined potentially significant associations between the presence of peripheral drusen and demographic cohorts. We found that there was no significant difference in the distribution of peripheral drusen observed in eyes segmented by age cohorts (Supplementary Table 5). Similarly, we also did not see a significant difference in the observation of peripheral drusen types segmented by age in patients diagnosed with late-stage AMD (Supplementary Table 6). When utilizing the CLMM, age was associated with an odds ratio of 1.03 (*p* = 0.110), meaning that a 1-year increase to Age was associated with a 3% increase to the odds that a patient will have more affected quadrants, holding all other factors constant. However, these findings were not statistically significant.

We finally examined whether there were significant trends of peripheral drusen observed in cohorts segmented by race. As seen in Supplementary Table 7, our assessment did not show any significant differences between peripheral drusen type observed in UWF by racial cohorts.

## Discussion

Our study found that peripheral drusen was commonly seen in the periphery of retinal patients through UWF imaging. Examining peripheral drusen through UWF by subtype, we found that hard drusen was the most common type of peripheral drusen. Our study found that peripheral hard drusen was associated with a diagnosis of AMD but did not find that all peripheral drusen types were associated with an AMD diagnosis, unlike the findings seen by some studies^1,2^.

While there have been previous investigations into examining peripheral drusen in UWF images, there has not been a validated system with which to differentiate between peripheral drusen sub-types^1,11,20^. In the present study we propose a new validated peripheral drusen grading system and provide a basic image library to assist others to distinguish types of peripheral drusen (Supplementary Fig. 2). Our study hoped to generate a list of common peripheral drusen types to enable a grading system that can be used by clinicians to better understand peripheral changes in AMD. We also developed a basic image guide to compare peripheral drusen deposits in the retina to improve their detection in the clinical setting.

AMD disease severity is partly graded by the accumulation of macular drusen deposits^5–7^. The present study focused on whether there was a similar association between AMD severity and the presence of peripheral drusen deposits. We found no association between peripheral drusen presence and the severity of AMD. Further investigation, using a prospective longitudinal study, will be required to see whether the presence of peripheral hard drusen a prognostic marker of AMD severity is. To our knowledge no previous papers have investigated the relationship between peripheral drusen and AMD severity.

Because age is also a well-known marker for AMD progression, we also examined whether there were differences in peripheral drusen profile by age and specifically in patients diagnosed with late-stage AMD (Supplementary Fig. 4). Our assessment did not show a significant presence of any kind of peripheral drusen when analyzed by age, both overall and in late-stage AMD. Our assessment also did not show a significant presence of any race-related trends with peripheral drusen. Future studies examining such demographic trends would greatly benefit with a greater sample size.

The present study was based on patients seen consecutively in retina clinics. Our study therefore has limitations regarding generalizability of conclusions to the general population. Also, as this was cross sectional study. As a result, it is unclear whether a particular peripheral drusen subtype would lead to increased severity of AMD with time. Thus, it would be ideal if our early findings were studied in more detail using a prospective long-term longitudinal study to see if patients ultimately have more severity with different subtypes of peripheral drusen. Finally, there are limitations in the ability of UWF pseudocolor imaging to precisely distinguish between drusen subtypes in the retinal periphery. We did not find an increase in drusen with age in our cohort. This is likely because we only counted drusen presence by quadrant. Past studies also suggest that the superior and inferior quadrants provide limited visibility of the retinal periphery, which could potentially lead to a misinterpretation of some images^21,22^. It would be helpful for future studies to utilize UWF OCT scans to better visualize different drusen subtypes in the retinal periphery in addition to the use of UWF pseudocolor fundus images.

In conclusion, the present study provides a perspective on the association between peripheral drusen and AMD and adds to the existing literature. In addition, the study provides a new validated grading system, with reference images, for peripheral drusen and it confirms the findings for previous studies that peripheral drusen can be associated with AMD.

### Supplementary Information

Supplementary Information.

## Data Availability

All relevant data is listed in this manuscript. Additional inquiries can be directed towards the corresponding author.
