# Supplementary material for: Developing an image-based grading scale for peripheral drusen to investigate associations of peripheral drusen type with age-related macular degeneration
Source: Sci Rep. 2024 Aug 29;14:20041. doi: 10.1038/s41598-024-70352-3 (PMC11358136; doi:10.1038/s41598-024-70352-3)
Supplement: Supplementary file 1 — Supplementary Information. [file 41598_2024_70352_MOESM1_ESM.pdf]

Supplementary Figure 1. Example image of the quadrants utilized to identify peripheral drusen in ultra-widefield pseudocolor fundus images.

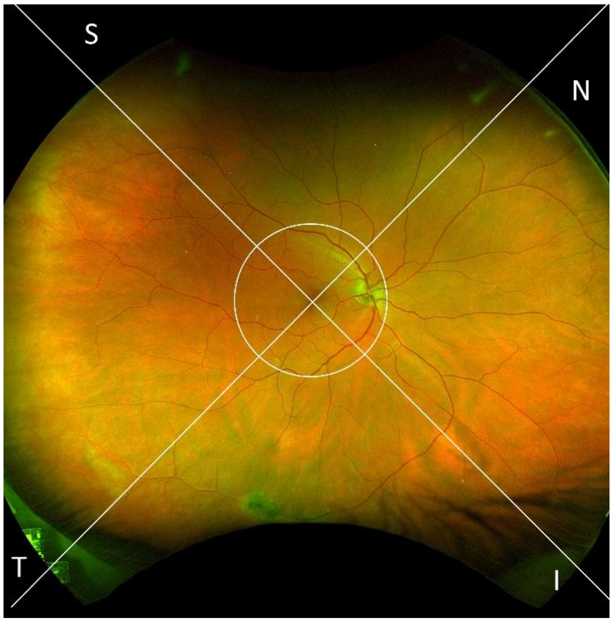

Supplementary Figure 2. Example illustrations of each type of peripheral drusen identified in the study by masked retinal specialists.

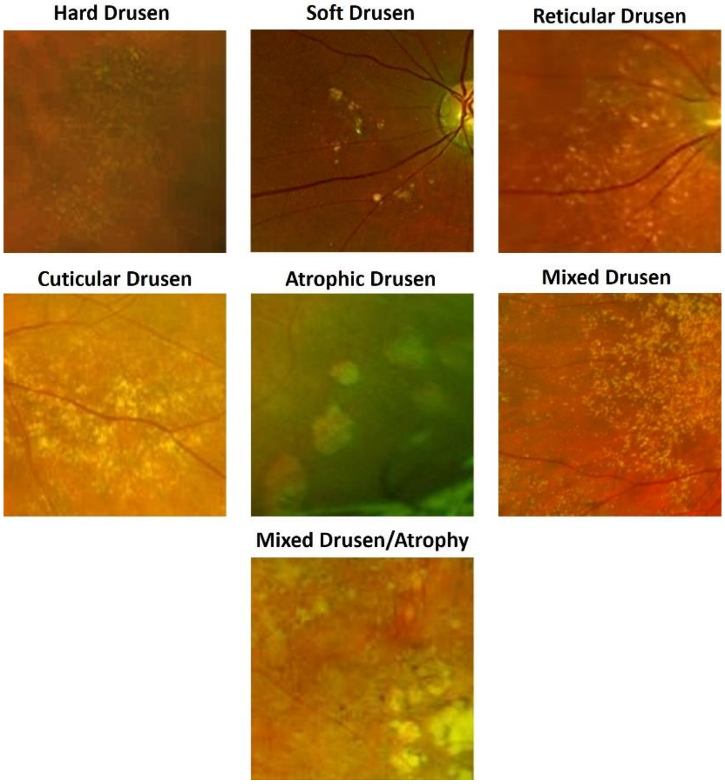

Supplementary Figure 3. Graph depicting eyes observed with each type of peripheral drusen by quadrant.

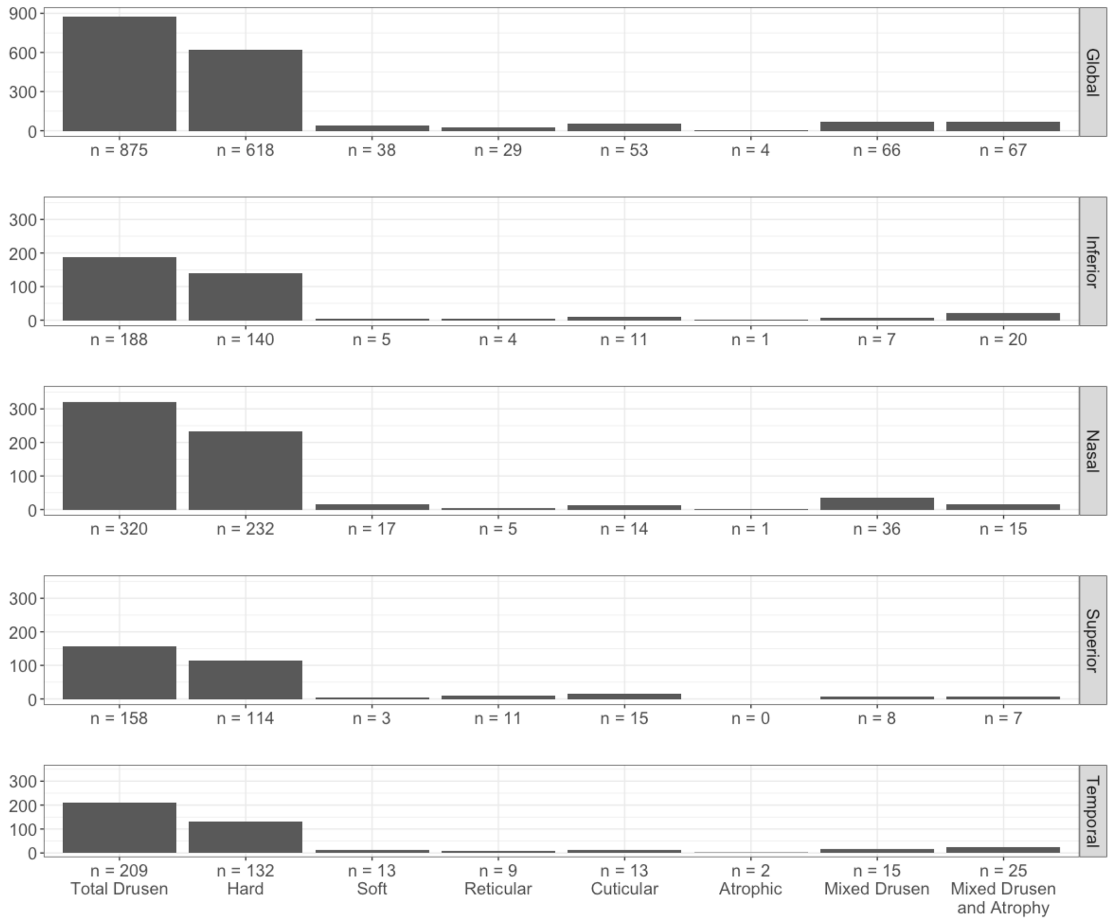

Supplementary Figure 4. Examples of eyes with late-stage AMD that did and did not exhibit peripheral drusen. Image A (left) is a patient with late-stage general atrophy (GA) AMD without peripheral drusen. Image B (right) is a patient with late-stage GA AMD who was observed with peripheral hard drusen in the superior, nasal, and inferior quadrants.

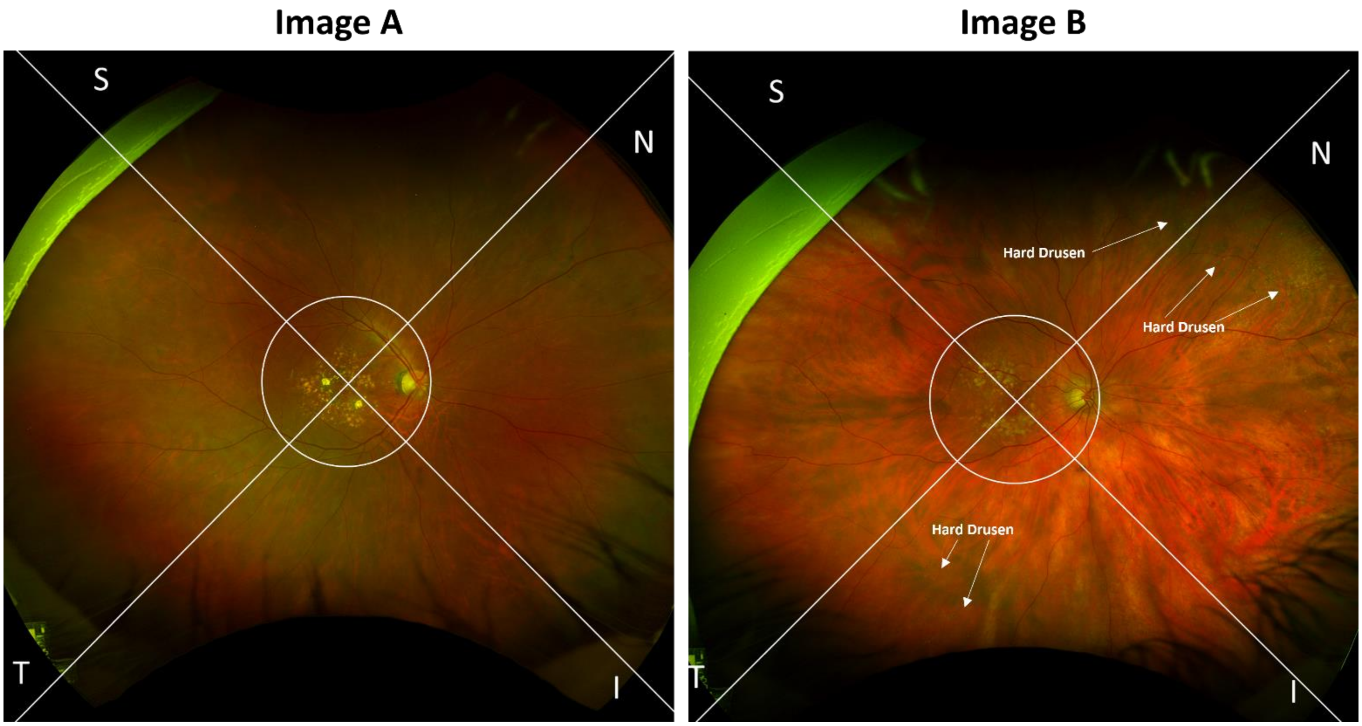

Supplementary Table 1. Summary of eyes excluded from the study.

Image Exclusion Criteria	Ungradable Eyes N = 2,861
**Ultra-widefield pseudocolor fundus images not passing quality screening**	
**Less than 60% peripheral visibility in at least one quadrant**	1,270 (44.4%)
**Lacks 4+ arteriole visibility in at least one quadrant**	191 (6.7%)
**No AMD per SD-OCT and no visible peripheral drusen per UWF**	1,388 (48.5%)
**Confounding UWF peripheral abnormalities**	
**Pan-retinal photocoagulation (PRP) &/or laser marks**	10 (0.35%)
**Asteroid hyalosis**	2 (0.07%)

Supplementary Table 2. Inter-rater agreement generated using Cohen's Kappa between the primary masked retinal specialist and the secondary evaluating 50 right and 50 left UWF images.

Quadrant	Kappa	p-value
Global	0.649	< 0.001
Superior	0.660	< 0.001
Nasal	0.579	< 0.001
Inferior	0.652	< 0.001
Temporal	0.622	< 0.001

Supplementary Table 3. Intra-rater agreement generated using Cohen's Kappa for the primary grader evaluating the same 50 right and 50 left UWF images one week apart.

Quadrant	Kappa	p-value
Global	0.922	< 0.001
Superior	0.947	< 0.001
Nasal	0.920	< 0.001
Inferior	0.893	< 0.001
Temporal	0.915	< 0.001

Supplementary Table 4. Outlines the number of eyes with different types of peripheral drusen across AMD staging.

	No AMD (n = 196)	Any AMD n = 284)	Early-stage AMD (n = 45)	Intermediate-stage AMD (n = 144)	Late-stage AMD (n = 95)	Late (Wet) (n = 66)	Late (GA) (n = 29)
Total Drusen							
Not Present	33 (16.8%)	93 (32.7%)	11 (24.2%)	51 (35.4%)	31 (32.6%)	22 (33.3%)	9 (31.0%)
Present	163 (83.2%)	191 (67.3%)	34 (75.6%)	93 (64.6%)	64 (67.4%)	44 (66.7%)	20 (69.0%)
Hard Drusen							
Not Present	65 (33.2%)	131 (46.1%)	16 (35.6%)	69 (47.9%)	46 (48.4%)	31 (47.0%)	15 (51.7%)
Present	131 (66.8%)	153 (53.9%)	29 (64.4%)	75 (52.1%)	49 (51.6%)	35 (53.0%)	14 (48.3%)
Soft							
Not Present	177 (90.3%)	275 (96.8%)	44 (97.8%)	138 (95.8%)	93 (97.9%)	65 (98.5%)	28 (96.6%)
Present	19 (9.7%)	9 (3.2%)	1 (2.2%)	6 (4.2%)	2 (2.1%)	1 (1.5%)	1 (3.4%)
Reticular							
Not Present	189 (96.4%)	276 (97.2%)	42 (93.3%)	141 (97.9%)	93 (97.9%)	64 (97.0%)	29 (100.0%)
Present	7 (3.6%)	8 (2.8%)	3 (6.7%)	3 (2.1%)	2 (2.1%)	2 (3.0%)	0 (0.0%)
Cuticular							
Not Present	185 (94.4%)	273 (96.1%)	44 (97.8%)	135 (93.8%)	94 (98.9%)	66 (100.0%)	28 (96.6%)
Present	11 (5.6%)	11 (3.9%)	1 (2.2%)	9 (6.2%)	1 (1.1%)	0 (0.0%)	1 (3.4%)
Atrophic							
Not Present	194 (99.0%)	282 (99.3%)	44 (97.8%)	143 (99.3%)	95 (100.0%)	66 (100.0%)	29 (100.0%)
Present	2 (1.0%)	2 (0.7%)	1 (2.2%)	1 (0.7%)	0 (0.0%)	0 (0.0%)	0 (0.0%)
Mixed Drusen							
Not Present	183 (93.4%)	259 (91.2%)	39 (86.7%)	136 (94.4%)	84 (88.4%)	60 (90.9%)	24 (82.8%)
Present	13 (6.6%)	25 (8.8%)	6 (13.3%)	9 (6.2%)	11 (11.6%)	6 (9.1%)	5 (17.2%)
Mixed Drusen and Atrophy							
Not Present	193 (98.5%)	255 (89.8%)	41 (91.1%)	134 (93.1%)	80 (84.2%)	56 (84.8%)	24 (82.8%)
Present	3 (1.5%)	29 (10.2%)	4 (8.9%)	10 (6.9%)	15 (15.8%)	10 (15.2%)	5 (17.2%)

Supplementary Table 5. The generalized linear mixed-effects and mixed ANOVA model was used to generate p-values examining associations between the presence of peripheral drusen and age cohorts.

	< Median Age (n = 232)	> Median Age (n = 248)	p-value
Total Drusen			
Not Present	56 (24.1%)	70 (28.2%)	0.457
Present	176 (75.9%)	178 (71.8%)	
Hard			
Not Present	91 (39.2%)	105 (42.3%)	0.556
Present	141 (60.8%)	143 (57.7%)	
Soft			
Not Present	215 (92.7%)	237 (95.6%)	0.209
Present	17 (7.3%)	11 (4.4%)	
Reticular			
Not Present	224 (96.6%)	241 (97.2%)	0.738
Present	8 (3.4%)	7 (2.8%)	
Cuticular			
Not Present	221 (95.3%)	237 (95.6%)	0.884
Present	11 (4.7%)	11 (4.4%)	
Atrophic			
Not Present	230 (99.1%)	246 (99.2%)	0.947
Present	2 (0.9%)	2 (0.8%)	
Mixed Drusen			
Not Present	218 (94.0%)	224 (90.3%)	0.153
Present	14 (6.0%)	24 (9.7%)	
Mixed Drusen and Atrophy			
Not Present	219 (94.4%)	229 (92.3%)	0.511
Present	13 (5.6%)	19 (7.7%)	

Supplementary Table 6. The generalized linear mixed-effects and mixed ANOVA model was used to generate p-values examining associations between the presence of peripheral drusen and age cohorts in patients diagnosed with late-stage AMD.

	< Median Age (n = 22)	> Median Age (n = 73)	p-value
Total Drusen			
Not Present	9 (40.9%)	22 (30.1%)	0.263
Present	13 (59.1%)	51 (69.9%)	

Hard			
Not Present	12 (54.5%)	34 (46.6%)	0.382
Present	10 (45.5%)	39 (53.4%)	
Soft			
Not Present	21 (95.5%)	72 (98.6%)	0.369
Present	1 (4.5%)	1 (1.4%)	
Reticular			
Not Present	22 (100.0%)	71 (97.3%)	0.439
Present	0 (0.0%)	2 (2.7%)	
Cuticular			
Not Present	22 (100.0%)	72 (98.6%)	0.999
Present	0 (0.0%)	1 (1.4%)	
Atrophic			
Not Present	22 (100.0%)	73 (100.0%)	0.999
Present	0 (0.0%)	0 (0.0%)	
Mixed Drusen			
Not Present	21 (95.5%)	63 (86.3%)	0.295
Present	1 (4.5%)	10 (13.7%)	
Mixed Drusen and Atrophy			
Not Present	19 (86.4%)	61 (83.6%)	0.792
Present	3 (13.6%)	12 (16.4%)	

Supplementary Table 7. The generalized linear mixed-effects and mixed ANOVA model was used to generate p-values examining associations between the presence of peripheral drusen and racial cohorts.

	African American or Black (n = 5 patients, 8 eyes)	Asian (n = 24 patients, 39 eyes)	Other or Mixed Race (n = 37 patients, 63 eyes)	White (n = 216 patients, 370 eyes)	p-value
Total Drusen					
Not Present	3 (37.5%)	12 (30.8%)	21 (33.3%)	90 (24.3%)	0.429
Present	5 (62.5%)	27 (69.2%)	42 (66.7%)	280 (75.7%)	
Hard					
Not Present	3 (37.5%)	24 (61.5%)	29 (46.0%)	140 (37.8%)	0.085
Present	5 (62.5%)	15 (38.5%)	34 (54.0%)	230 (62.2%)	
Soft					
Not Present	8 (100.0%)	33 (84.6%)	61 (96.8%)	350 (94.6%)	0.074
Present	0 (0.0%)	6 (15.4%)	2 (3.2%)	20 (5.4%)	
Reticular					

Not Present	8 (100.0%)	37 (94.9%)	58 (92.1%)	362 (97.8%)	0.119
Present	0 (0.0%)	2 (5.1%)	5 (7.9%)	8 (2.2%)	
Cuticular					
Not Present	8 (100.0%)	34 (87.2%)	62 (98.4%)	354 (95.7%)	0.143
Present	0 (0.0%)	5 (12.8%)	1 (1.6%)	16 (4.3%)	
Atrophic					
Not Present	8 (100.0%)	38 (97.4%)	63 (100.0%)	367 (99.2%)	0.572
Present	0 (0.0%)	1 (2.6%)	0 (0.0%)	3 (0.8%)	
Mixed Drusen					
Not Present	8 (100.0%)	35 (89.7%)	60 (95.2%)	339 (91.6%)	0.617
Present	0 (0.0%)	4 (10.3%)	3 (4.8%)	31 (8.4%)	
Mixed Drusen and Atrophy					
Not Present	8 (100.0%)	39 (100.0%)	59 (93.7%)	342 (92.4%)	0.398
Present	0 (0.0%)	0 (0.0%)	4 (6.3%)	28 (7.6%)	
